# Expression Pattern of Fatty Acid Binding Proteins in Celiac Disease Enteropathy

**DOI:** 10.1155/2015/738563

**Published:** 2015-08-05

**Authors:** Natalia M. Bottasso Arias, Marina García, Constanza Bondar, Luciana Guzman, Agustina Redondo, Nestor Chopita, Betina Córsico, Fernando G. Chirdo

**Affiliations:** ^1^Instituto de Investigaciones Bioquímicas de La Plata (INIBIOLP), CCT CONICET, Facultad de Ciencias Médicas, Universidad Nacional de La Plata, 1900 La Plata, Argentina; ^2^Departamento de Ciencias Biológicas, Instituto de Estudios Inmunológicos y Fisiopatológicos (IIFP), UNLP-CONICET, Facultad de Ciencias Exactas, Universidad Nacional de La Plata, 1900 La Plata, Argentina; ^3^Laboratorio de Inmunología de Enfermedades Respiratorias, Instituto de Medicina Experimental (IMEX), CONICET, Academia Nacional de Medicina, C1425AUM Buenos Aires, Argentina; ^4^Servicio de Gastroenterología, Hospital de Niños “Sor María Ludovica”, 1900 La Plata, Argentina; ^5^Servicio de Gastroenterología, Hospital “San Martín”, 1900 La Plata, Argentina

## Abstract

Celiac disease (CD) is an immune-mediated enteropathy that develops in genetically susceptible individuals following exposure to dietary gluten. Severe changes at the intestinal mucosa observed in untreated CD patients are linked to changes in the level and in the pattern of expression of different genes. Fully differentiated epithelial cells express two isoforms of fatty acid binding proteins (FABPs): intestinal and liver, IFABP and LFABP, respectively. These proteins bind and transport long chain fatty acids and also have other important biological roles in signaling pathways, particularly those related to PPAR*γ* and inflammatory processes. Herein, we analyze the serum levels of IFABP and characterize the expression of both FABPs at protein and mRNA level in small intestinal mucosa in severe enteropathy and normal tissue. As a result, we observed higher levels of circulating IFABP in untreated CD patients compared with controls and patients on gluten-free diet. In duodenal mucosa a differential FABPs expression pattern was observed with a reduction in mRNA levels compared to controls explained by the epithelium loss in severe enteropathy. In conclusion, we report changes in FABPs' expression pattern in severe enteropathy. Consequently, there might be alterations in lipid metabolism and the inflammatory process in the small intestinal mucosa.

## 1. Introduction

Celiac disease (CD) is a chronic immune-mediated enteropathy with 1% worldwide prevalence. CD is triggered by ingestion of a group of proteins, commonly known as gluten, from wheat, barley, and rye, in genetically susceptible individuals [1, 2]. CD is a polygenic disorder and certain HLA alleles are the most important genetic factors involved. Almost all of the patients carry the HLA variants HLA-DQ2 (DQA1^*∗*^05:01, DQB1^*∗*^02:01), HLA-DQ8 (DQA1^*∗*^03, DQB1^*∗*^03:02), and HLA-DQ2 (DQA1^*∗*^02:01, DQB1^*∗*^02:02) [3]. The role of HLA molecules was clearly established when gluten-specific T cells were isolated from small intestine of CD patients at diagnosis. These T cells are IFN*γ* producers and are restricted to HLA-DQ2 or HLA-DQ8 molecules [3, 4]. Though CD presents the highest association with particular HLA alleles in comparison with other diseases, these genes only account for 40–50% of the genetic susceptibility. Therefore, HLA is considered a necessary, but not a sufficient, factor for CD development. In recent genetic studies, 39 non-HLA loci were found to be susceptibility factors for CD [5].

CD enteropathy is commonly limited to proximal small intestine where the characteristic histological findings are villous atrophy, crypt hyperplasia, and lymphocytic infiltration. These histological changes cause loss of intestinal function and malabsorption syndrome. Mechanisms of both innate and adaptive immunity are involved in the damage to the small intestinal mucosa [1, 2].

Currently, CD diagnosis is based on clinical evaluation, positive serology (anti-transglutaminase 2, anti-deamidated gluten peptides, and anti-endomysium antibodies), and the histological examination of a small intestine biopsy. Dietary exclusion of gluten proteins (gluten-free diet, GFD) restores the histology of the intestinal mucosa, reverts the symptoms, and is considered as complementary information in the diagnosis of CD [6]. Though serological tests present high analytical efficiency, CD diagnosis is still based on the assessment of the histology of intestinal biopsies. New noninvasive tools for diagnosis and follow-up of CD patients are needed.

Intestinal and liver fatty acid binding protein (IFABP and LFABP, resp.) have been reported as markers of intestinal epithelial damage in mesenteric thrombosis, necrotizing colitis, and celiac disease [7–10]. The fatty acid binding protein (FABP) family comprises 9 isoforms of small cytosolic proteins (14-15 kDa) expressed in different tissues, where more than one type of FABP can be found [11]. These proteins bind and transport long chain fatty acids (among other hydrophobic ligands). Specifically, IFABP and LFABP have been suggested by* in vitro* studies to be involved in lipid uptake from the intestinal lumen into the enterocyte [12, 13].

IFABP and LFABP are highly expressed in the enterocyte, representing 1-2% of the total cytosolic proteins [14]. Moreover, the mRNAs of IFABP and LFABP are the most abundant translatable RNA sequences in the gut epithelium [15]. Nevertheless, their individual functions in the intestine have not been fully elucidated. Several lines of evidence indirectly suggest they may perform different functions within the same cell type. For example, although all the FABPs have a highly conserved tertiary structure, containing a 10-strand beta-barrel where ligands are bound [11, 16], IFABP binds a single fatty acid per molecule whereas LFABP can bind up to two fatty acids and other hydrophobic ligands [17, 18]. Fatty acid binding specificity and mechanism of transfer to membranes are also different for IFABP and LFABP [19, 20].

Studies by immunofluorescence showed that IFABP and LFABP are expressed in the epithelium of normal small intestine [21]. Differentiated enterocytes abundantly express IFABP and LFABP, and the latter is produced in 40 to 50 times higher concentration [8, 22]. Due to enterocyte damage occurring in CD enteropathy, LFABP and IFABP are released into peripheral blood. As a consequence, levels of LFABP and IFABP were found significantly elevated in circulation in untreated CD patients compared to nonceliac controls [10, 23–25].

The putative participation of intestinal FABPs in CD deserves further exploration for at least two main reasons. First, other FABPs have been found to participate as mediators in inflammatory processes in the tissues where they are expressed [26–28]. Second, the alteration of the enterocyte epithelia interferes with the absorption of nutrients and this could lead to modifications in intracellular lipid transport, proteins expression, and/or function.

With the aim of exploring these possibilities, after determining the serum levels of IFABP in a local population of untreated CD patients compared to nonceliac controls, we analyzed the expression pattern of IFABP and LFABP at protein and mRNA levels in human small intestine in normal and enteropathy tissues.

## 2. Materials and Methods

### 2.1. Ethics Statements

Duodenal biopsies and blood samples were obtained from pediatric and adult patients during routine procedures to diagnose CD at the Gastroenterology Units of Sor Maria Ludovica Hospital and HIGA San Martin from La Plata, Buenos Aires, Argentina, respectively. Patients or their guardians provided a written informed consent to participate in this study. The present study was approved by the Ethical Committees of the Sor Maria Ludovica Hospital and HIGA San Martin from La Plata, Argentina.

### 2.2. Patients and Samples

CD diagnosis was achieved by evaluation of clinical presentation, histological examination, and serological analysis. All pediatric and adult celiac patients had classical presentation, with signs and symptoms of malabsorption including chronic diarrhea, abdominal pain, distension, and failure to thrive or weight loss. Serological studies included determination of IgG and IgA anti-transglutaminase 2 antibodies, IgG and IgA anti-deamidated gluten peptides antibodies. In all the cases, positive serology was considered when antibody levels were, at least, twice the cut-off level.

For diagnostic purposes, three biopsy pieces were taken from the second portion of the duodenum and evaluated by an expert pathologist.

Altogether, this study included CD patients with total or partial villus atrophy, classical clinical manifestations, and positive serology.

Patients on gluten-free diet (GFD) presented histological recovery and negative serological markers for CD. Nonceliac individuals who suffered from other gastrointestinal conditions, primarily dyspepsia, and presented negative CD serology and normal duodenal histology were also included in this study. The diagnosis of inflammatory bowel disease was confirmed by established clinical, endoscopic, and histological criteria. In this study, samples from a pediatric patient suffering from ulcerative colitis and six from adult patients (4 with ulcerative colitis and 2 with Crohn's disease) were included. Crohn's disease patients had no ileal involvement. All samples of IBD patients were taken at diagnosis and they did not receive any treatment at the time of sample collection.

Serum samples were collected from 42 nonceliac controls (16 pediatric cases, 26 adults), 40 untreated CD patients (17 pediatric cases, 23 adults), 9 patients on gluten-free diet (GFD) (3 pediatric cases, 6 adults), and 7 intestinal bowel disease (IBD) patients (1 pediatric case, 6 adults).

Duodenal samples were obtained from 27 pediatric patients (13 nonceliac controls, 14 CD patients) and 15 adult individuals (9 nonceliac controls, 6 CD patients). Samples were stabilised using RNAlater (Ambion, cat. AM7020) and stored at −80°C until processing for gene expression analysis.

### 2.3. Assessment of Serum Levels of IFABP

Determination of IFABP serum levels was performed using an ELISA kit (HyCult Biotech, HK406) following the instructions provided by the manufacturer. Serum samples from nonceliac controls, treated and untreated celiac disease patients, and IBD patients were analyzed. Values are expressed as mean ± standard deviation (s.d.) of the mean.

### 2.4. Production of Anti-FABPs Polyclonal Antibodies

We analyzed the expression of human FABPs in duodenal samples using rabbit polyclonal antibodies raised against rat FABPs. Sequence and structure of rat and human FABPs have been well established. The 132-residue rat and human IFABPs have 82% amino acids' sequence identity [29] leading to a high degree of homology (83%) and cross-reactivity between human and rat LFABP [30]. Recombinant rat IFABP and LFABP were produced in* E. coli BL21 (DE3)* cells transformed pET11d-IFABP, pET11a-LFABP and purified as described elsewhere [20, 31], performing exclusion chromatography (Sephadex G-50, Pharmacia Biotech Inc.), followed by anion exchange chromatography (DE-52, Whatman) and finally delipidation using a Lipidex-1000 column. SDS-PAGE electrophoresis followed by Coomassie Brilliant Blue R250 (Thermo Scientific) staining was used to assess FABP purity. Purified IFABP and LFABP were used to immunize rabbits according to the standard protocol [32]. Reactivity of polyclonal antibodies from immunized rabbits was assessed by western blotting and ELISA using the recombinant proteins. Western blot was developed using a chemiluminescent HRP substrate (Thermo, cat. 34080).

### 2.5. Analysis of IFABP and LFABP Expression in Small Intestine

Sections of paraffin-embedded small intestine biopsies were obtained using a Leica SM 2000R microtome; subsequently they were rehydrated and Citra solution (Biogenex, cat. HK086-9K) was used for antigen retrieval. Sections were stained with anti-IFABP or anti-LFABP rabbit polyclonal antibodies (dilutions used: LFABP 1/200–1/400, IFABP 1/20–1/50). Afterwards, they were incubated with anti-rabbit IgG conjugated with Alexa 488 (Life Technologies, cat. A-21206). Nuclei were stained with propidium iodide or DAPI. Confocal fluorescence microscopy analysis was performed in a SP5 Leica confocal microscope. Immunofluorescence analysis was performed in a Nikon Eclipse E400 fluorescence microscope.

### 2.6. Quantitative PCR Analysis

Total RNA was isolated from whole biopsy samples using an RNA Spin Mini kit (GE Healthcare, cat. 25-0500-72). Reverse transcription was performed using 1 *µ*g of total RNA. MML-V polymerase and random primers were obtained from Molecular Probes Inc., Invitrogen (Carlsbad, CA, USA). Quantitative Real Time PCR was performed in an IQ Cycler from BioRad using SYBR green mix (Invitrogen, cat. 11761-100) and specific primers for the genes of interest: IFABP forward AGCACTTGGAAGGTAGACCG, IFABP reverse CCCCTGAGTTCAGTTCCGTC; LFABP forward AGCTCTATTGCCACCATGAGTTTCT, LFABP reverse AACTGAACCACTGTCTTGACTTTCTC; *β*-actin forward ATGGGTCAGAAGTCCTATGTG, *β*-actin reverse CTTCATGAGGTAGTCAGTCAGGTC; villin forward CTACACCACACAGATGGATGACTTC, villin reverse GACATCTCTACCTCTCCAGCTACCA. The comparative Ct method was used to quantify IFABP and LFABP transcripts normalizing alternatively with *β*-actin or villin. Values are expressed as mean ± standard deviation (s.d.) of the mean.

### 2.7. Statistical Analysis

Both statistical evaluation of serum levels of IFABP by ELISA and quantitative PCR analysis of IFABP and LFABP mRNA levels were performed by the Mann-Whitney *U* test.

## 3. Results

### 3.1. IFABP Serum Levels

**Figure 1:**
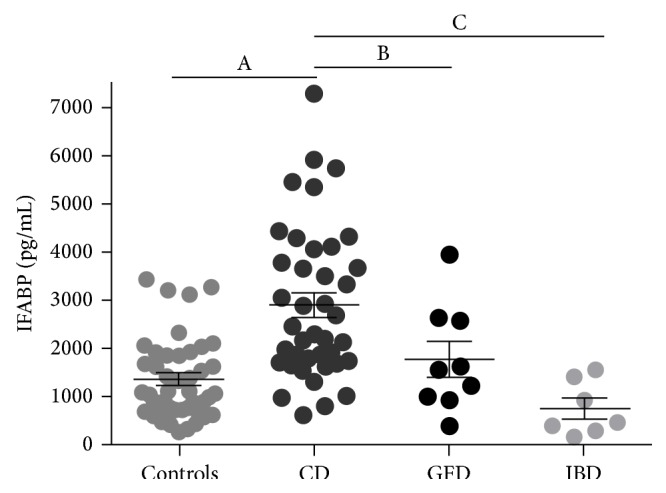
Increased levels of IFABP in serum samples of active CD. IFABP serum levels (pg/mL) were assessed by commercial quantitative ELISA. Serum samples from adults and pediatric patients were plotted together since no differences between both populations were observed. Serum samples from controls (*n* = 42), untreated CD patients (*n* = 40), CD patients on gluten-free diet (GFD) (*n* = 9), and intestinal bowel disease (IBD) patients (*n* = 7) were analyzed. Values are expressed as mean ± standard deviation (s.d.) of the mean. Representative experiments were analyzed statistically using the Mann-Whitney *U* test. (A) *p* < 0.0001; (B) *p* = 0.0002; (C) *p* = 0.0264.

The concentration of IFABP in serum samples was determined by a commercial quantitative ELISA kit. Untreated CD patients presented significant higher levels of IFABP in serum compared to treated CD patients, non-CD controls, or IBD patients. Because there was no difference between samples from pediatric and adult populations, the results obtained from both populations were depicted together. No differences were found between non-CD controls, CD patients on GFD, and IBD patients (Figure 1). The assessment of IFABP concentration in serum samples showed statistical differences for samples from CD patients at diagnosis (mean value 2898.89 pg/mL, range 616.83–7295.95 pg/mL), non-CD controls (mean value 1356.49 pg/mL, range 256.51–3433.33 pg/mL), CD patients on GFD (mean value 1766.84 pg/mL, range 391.42–3955.88 pg/mL), and IBD patients (mean value 744.92 pg/mL, range 165.89–1558.2 pg/mL).

Serum IFABP levels might serve to discriminate CD patients from non-CD controls as described by previous reports [10, 23–25].

Similar IFABP levels in serum from patients on GFD and controls were observed, suggesting that IFABP may also be a biomarker to follow up the response to treatment. Remarkably, IFABP concentration in serum samples from IBD patients was similar to the values observed in non-CD controls. These results suggest that the increase of IFABP concentration in serum is a specific finding in CD enteropathy, which is limited to the proximal small intestine.

### 3.2. Expression of LFABP and IFABP in Human Duodenum

**Figure 2:**
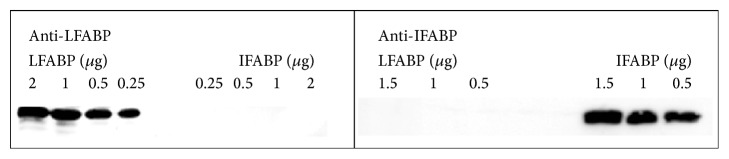
Assessment of reactivity of the rabbit polyclonal antibodies. Western blot analysis was performed using recombinant purified LFABP (protein loaded: 0.25–2 *μ*g) and IFABP (protein loaded: 0.25–2 *μ*g). Rabbit anti-LFABP and anti-IFABP polyclonal antibodies were incubated at 1/6000 and 1/20000 dilutions, respectively.

In order to evaluate the expression pattern of LFABP and IFABP in human duodenum by immunofluorescence techniques, rabbit polyclonal antibodies were raised by inoculating purified recombinant rat IFABP or LFABP. Western blot analysis showed that each of the polyclonal antibodies produced in our laboratory specifically reacts with the protein used as antigen. No cross-reactivity was observed between IFABP and LFABP (Figure 2). These antibodies proved to be useful for detecting human IFABP and LFABP. This reactivity is based on the homology of these human and rat FABPs. Specific labeling was verified on duodenal sections from healthy control and enteropathy samples using serum from an unimmunized rabbit (see Supplementary Figure 1 in the Supplementary Material available online at http://dx.doi.org/10.1155/2015/738563).

**Figure 3:**
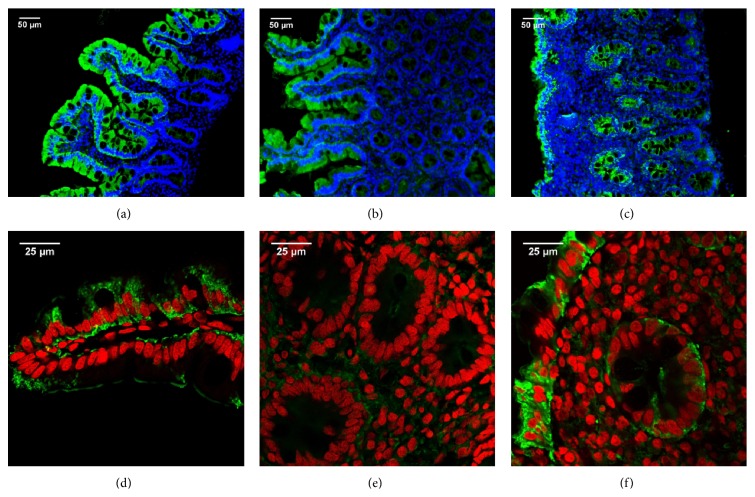
LFABP expression in human small intestine. Indirect immunofluorescence analysis using anti-LFABP polyclonal antibodies (Alexa 488, green) and nuclei stained with DAPI (blue). Representative staining in duodenal sections of non-CD control ((a) and (b)) and CD patient at diagnosis (c) (magnification 20x). Confocal fluorescence microscopy using anti-LFABP polyclonal antibodies (Alexa 488, green) and nuclei stained with propidium iodide (red). Representative staining in duodenal sections of non-CD control ((d) and (e)) and CD patient at diagnosis (f) (magnification 63x + 1.7 zoom). Healthy tissue ((a), (b), (d), and (e)) shows LFABP expression in the villi enterocytes. Severe enteropathy ((c) and (f)) shows LFABP expression in the epithelium as well as the crypts closer to the epithelium.

The expression of LFABP was analyzed in sections of duodenal biopsies from healthy controls and CD patients at diagnosis. Conventional indirect immunofluorescence analysis showed that LFABP is abundantly expressed in enterocytes from the villi but not in the crypts in healthy tissue (Figures 3(a) and 3(b)). In duodenal samples from CD patients LFABP was observed in enterocytes and remarkably in the crypts (Figure 3(c)). LFABP expression was more intense in the crypts closer to the epithelium fading away towards the* muscularis mucosae*. Similar findings were observed by fluorescence confocal microscopy (Figures 3(d) and 3(e)). Fully differentiated enterocytes showed homogeneous cytoplasmic staining for LFABP in the epithelium from tissues of both non-CD controls and CD patients. Crypts closer to the epithelium were also stained in severe enteropathy (Figures 3(c) and 3(f)).

**Figure 4:**
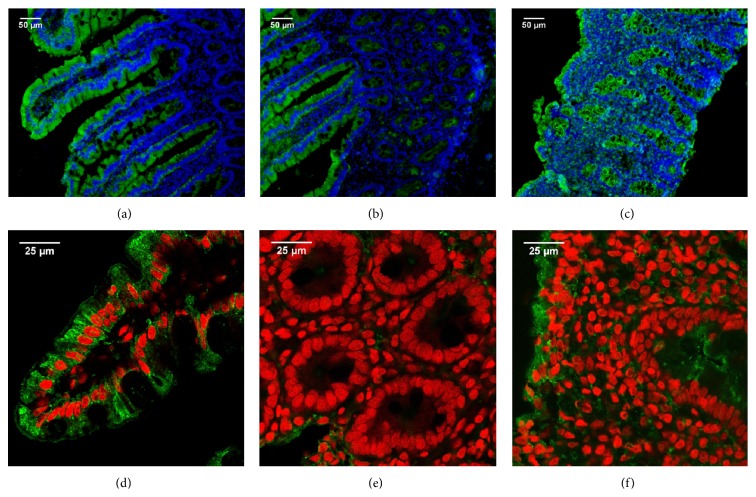
IFABP expression in human small intestine. Indirect immunofluorescence analysis using anti-IFABP polyclonal antibodies (Alexa 488, green) and nuclei stained with DAPI (blue). Representative staining in duodenal sections of non-CD control ((a) and (b)) and CD patient at diagnosis (c) (magnification 20x). Confocal fluorescence microscopy using anti-IFABP polyclonal antibodies (Alexa 488, green) and nuclei stained with propidium iodide (red). Representative staining in duodenal sections of non-CD control ((d) and (e)) and CD patient at diagnosis (f) (magnification 63x + 1.7 zoom). Healthy tissue ((a), (b), (d), and (e)) shows IFABP expression in the villi enterocytes. Severe enteropathy ((c) and (f)) shows IFABP expression in the epithelium as well as the crypts.

The analysis of IFABP expression by conventional immunofluorescence microscopy showed bright staining in enterocytes but also very faint staining in some crypts of small intestine from nonceliac controls (Figures 4(a) and 4(b)). Enterocytes in the remaining epithelium and in the crypts were strongly stained in duodenal samples from CD patients at diagnosis (Figure 4(c)). Confocal fluorescence microscopy analysis showed homogeneous staining for IFABP in the enterocytes in healthy small intestine (Figures 4(d) and 4(e)). IFABP expression was observed in enterocytes in the damaged epithelium as well as the crypts in small intestine of untreated CD patients (Figure 4(f)).

### 3.3. LFABP and IFABP mRNA Levels in Small Intestine

The expression of LFABP and IFABP mRNA in duodenal tissue was assessed by quantitative PCR using *β*-actin as housekeeping gene.

**Figure 5:**
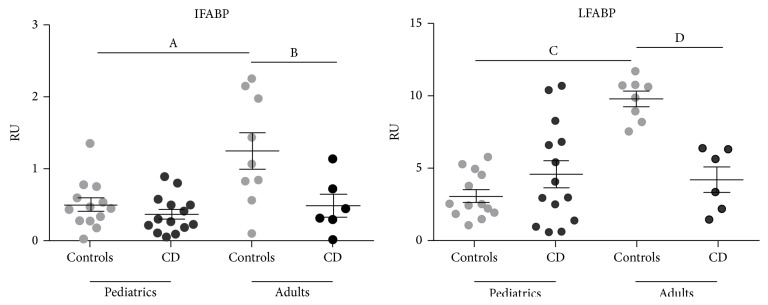
mRNA levels of LFABP and IFABP assessed by quantitative PCR using *β*-actin as housekeeping gene. Quantitative PCR analysis was performed in whole duodenal biopsies from adult and pediatric populations of healthy non-CD controls and CD patients at diagnosis. Pediatric samples: controls (*n* = 13), CD patients (*n* = 14). Adult samples: controls (*n* = 9 for IFABP, *n* = 8 for LFABP), CD patients (*n* = 6). Results were plotted as relative unit (RU), using *β*-actin as housekeeping gene. Values are expressed as mean ± standard deviation (s.d.) of the mean. Representative experiments were analyzed statistically using the Mann-Whitney *U* test. (A) *p* = 0.0047; (B) *p* = 0.0423; (C, D) *p* < 0.0001.

In non-CD controls, higher mRNA levels for both LFABP and IFABP were observed in adult than in pediatric small intestine (Figure 5). mRNA levels of LFABP were higher than those of IFABP. Strikingly, the expression of LFABP and IFABP was lower in untreated adult CD patients compared with non-CD controls. This difference was not observed in pediatric population.

FABPs' expression pattern, previously observed by immunofluorescence studies, suggests that an intense synthesis process occurs in fully differentiated enterocytes located in the villi. In severe enteropathy, these FABPs are also produced in the crypt enterocytes. However, a reduction in the mRNA levels in tissues showing severe enteropathy was observed.

**Figure 6:**
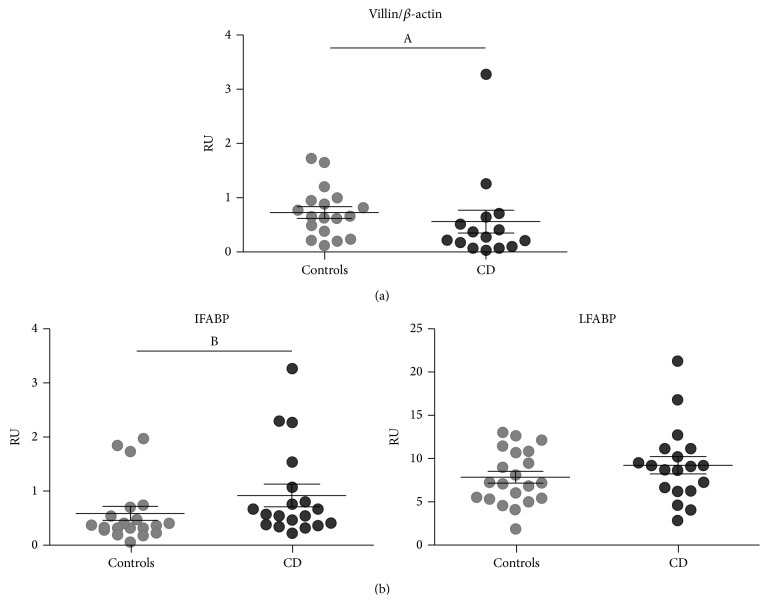
mRNA levels assessed by quantitative PCR of (a) villin using *β*-actin as housekeeping gene and (b) LFABP and IFABP using villin as housekeeping gene. Quantitative PCR analysis was performed in whole duodenal biopsies from adult and pediatric populations of healthy non-CD controls and CD patients at diagnosis. mRNA relative levels from adults and pediatric patients were plotted together since no differences between both populations were observed. (a) mRNA levels of villin using *β*-actin as housekeeping gene. Pediatric samples: controls (*n* = 10), CD patients (*n* = 9). Adult samples: controls (*n* = 8), CD patients (*n* = 6). (b) mRNA levels of LFABP and IFABP using villin as housekeeping gene. Pediatric samples: controls (*n* = 12), CD patients (*n* = 13). Adult samples: controls (*n* = 8), CD patients (*n* = 6). Results were plotted as relative unit (RU). Values are expressed as mean ± standard deviation (s.d.) of the mean. Representative experiments were analyzed statistically using the Mann-Whitney *U* test. (A) *p* = 0.0374; (B) *p* = 0.0294.

The small intestine in untreated CD patients shows relevant histological changes, particularly loss of epithelium and enlargement of* lamina propria*. These changes can be described as a reduction in the ratio of number of cells from epithelium versus cells from* lamina propria*. To estimate this variation, we focused our attention on the expression of villin, which is limited to differentiated enterocytes. Thus, the use of villin as housekeeping gene allows a better evaluation of changes in mRNA levels of proteins specifically expressed in the enterocytes [33]. As expected, the severe histological alterations observed in severe enteropathy were accompanied by changes at mRNA levels of villin compared with *β*-actin. Villin mRNA levels were reduced in severe enteropathy compared with normal tissue (Figure 6(a)).

Therefore, we decided to use villin as housekeeping gene to reevaluate the changes in the expression of FABPs in duodenal samples. We found that mRNA levels of both LFABP and IFABP were higher in samples with severe enteropathy compared with healthy tissue when villin was used as housekeeping gene, though a significant difference was only observed for IFABP. LFABP mRNA levels were higher than IFABP mRNA levels as described above (Figure 6(b)).

Altogether, these results indicate that in CD FABPs expression is actually increased inside enterocytes (significantly for IFABP) although their mRNA levels are lower when evaluating the whole intestinal sample due to the reduction of enterocyte to* lamina propria* cells ratio.

## 4. Discussion

CD is an immune-mediated enteropathy triggered by dietary gluten peptides in genetically susceptible individuals. The histological findings range from increased number of intraepithelial lymphocytes and a minor reduction in the villus height/crypt depth ratio up to total villus atrophy. The mechanism of mucosal damage is still not completely understood [2, 3].

Clinical evaluation, serology, and histology determine the diagnosis of CD. However, diagnosis cannot be established in some cases due to the lack of concordance between serology and histology [34, 35]. In addition, minor changes in the histology are frequently referred to as unspecific findings. Thus, a large number of cases may remain undiagnosed [36, 37]. HLA typing by identifying the presence of the HLA class II susceptibility alleles (HLA-DQ2/DQ8) is a complementary test with high negative predictive value and is useful as exclusion criteria. However, the high cost of this technique precludes its use in the routine clinical practice.

Noninvasive tests using markers for intestinal permeability have been used to evaluate the integrity of the gut mucosa [38, 39]. However, these tests are unspecific because many different conditions alter intestinal permeability. There is a current need for new tests for the identification of absorption alterations in the intestinal mucosa [40]. Thus, the search for noninvasive tests based on the evaluation of biomarkers that specifically detect CD or characterize its active stage is a promising tool for screening strategies, complementary information when diagnosis is controversial, or treatment follow-up. We recently showed that the inflammatory chemokine CXCL10 is abundantly produced in duodenal mucosa in untreated celiac patients, and also significant higher levels of circulating CXCL10 were found in untreated CD patients. Concentration of CXCL10 in serum returns to basal levels in patients on gluten-free diet. Though further studies evaluating large number of samples are required, these findings point to CXCL10 as a biomarker for the evaluation of CD [41]. Additionally, there are new proposed biomarkers of intestinal epithelial cell damage such as Reg-3*α* [42].

Enterocyte FABPs are proposed for binding and trafficking long chain fatty acids absorbed from the dietary lipids to different cell fates ranging from mitochondrial beta oxidation, regulation of gene expression in the nuclei, and chylomicron synthesis, among others [43]. Thus, it is possible to assume that in malabsorption syndromes, such as CD, these proteins may play a relevant role.

The measurement of cytosolic enterocyte proteins in peripheral blood has also been shown to be useful for estimating enterocyte damage [22]. IFABP and LFABP are highly expressed in enterocytes [21]. IFABP has also been proposed as a differentiation marker to evaluate intestinal maturation in preterm neonates [44]. Enterocytes on the top of the villi are the initial site of cell destruction in numerous intestinal diseases. Thus, high serum levels of FABPs may correlate with a higher rate of enterocyte damage. Serum IFABP was used as a biomarker to detect active CD. Since it is highly expressed in the villi tip, Vreugdenhil et al. [24] proposed IFABP as an early marker of intestinal damage in CD. In addition, as the expression of IFABP is limited to the intestinal epithelium, this protein is a better marker of intestinal damage than LFABP which is also expressed in other tissues [10]. The concentration of IFABP in serum samples also correlates with both anti-transglutaminase 2 IgA antibody levels and the severity of villous atrophy in CD patients at the time of diagnosis [23–25]. In addition, IFABP determination may have an advantage over anti-TG2 given that IFABP's half-life is shorter and could reflect rapid changes at mucosal level. IFABP values diminished to normalization in CD patients after GFD for at least a year, independently of initial antibodies and IFABP levels. Nevertheless, all the studies mentioned above reported the use of different cut-off values for IFABP levels in plasma. Therefore, with the aim of using the determination of serum IFABP in clinical practice, studies involving a large number of samples and validated procedures are needed.

Similarly to reported studies, in this work we observed higher concentrations of IFABP in serum samples of CD patients at the time of diagnosis compared to samples from patients on GFD or controls. Thus, the analysis of a local population replicated previous reports [10, 23–25] and showed that determination of the concentration of IFABP in serum may be used as a noninvasive test in diagnosis and GFD follow-up. In addition, our pilot study showed significantly higher IFABP levels in serum of CD patients compared to samples from IBD patients suggesting the potentiality of IFABP as a biomarker which may help in clinical practice to discriminate between samples from CD and IBD patients. To this end, further evaluation using a large number of cases is needed.

The use of circulating IFABP as a specific biomarker of small intestinal enterocytes damage was also suggested by Wiercinska-Drapalo et al. [45]. Authors found high levels of IFABP in serum from a group of ulcerative colitis patients suffering from ileitis, an extended inflammatory process in the terminal small intestine. This finding was associated with a diagnostic value of serum IFABP released from damaged enterocytes of the terminal small intestine.

In addition, analysis of IFABP urinary levels has been proposed as predictive biomarker of necrotising enterocolitis. Detection of IFABP in urine samples, as a consequence of its release from small intestine enterocytes, is useful for diagnosis since it may anticipate the rapid progression of this disease [46].

In agreement with a previous report [21], in this work we observed that LFABP and IFABP are highly expressed in the enterocytes of small intestine in healthy tissue. However, some differences in the pattern of expression were evidenced. Whereas Levy et al. [21] described expression of LFABP and IFABP in the crypts, we observed no staining for LFABP and a very faint staining for IFABP in the crypts from proximal duodenum. These differences are probably due to differences in the immunofluorescence techniques used, mainly the quality of the images obtained, and secondly the intestinal segments investigated in each study.

Our work revealed an important change in the expression of both FABPs when samples with severe enteropathy were analyzed. Remarkably, an intense crypt staining for both FABPs was observed. The staining for LFABP was more intense in crypts located close to the epithelium whereas IFABP showed an intense staining in all crypts, close to and far away from the remaining epithelium. This was a characteristic finding in tissues showing severe enteropathy from CD patients at the time of diagnosis. To our knowledge, this is the first description of this change in FABPs' expression pattern. The increased expression of FABPs in the immature enterocytes in the crypts may reflect an accelerated developmental program of enterocytes. Highly differentiated enterocytes are unable to replace the lost epithelia as a consequence of the extended damage process. Therefore, LFABP and IFABP, which are normally expressed in fully differentiated enterocytes from the small intestine, may appear earlier in the crypt enterocytes due to a faster developmental program.

Next, we evaluated the mRNA expression of LFABP and IFABP in small intestine using *β*-actin as housekeeping gene. Higher expression of both LFABP and IFABP was observed in control adult small intestine compared to control pediatric samples (Figure 5). By western blot analysis, Levy et al. [21] did not observe changes in the expression of FABPs at protein level in normal pediatric and adult jejunum. However, a quantitative analysis was not performed in this study.

Strikingly, the analysis of samples from adult CD patients at the time of diagnosis showed a significant reduction in the mRNA levels of LFABP and IFABP compared to healthy tissue. On the other hand, pediatric samples showed no difference in mRNA levels between CD patients and control for either protein (Figure 5).

Since qPCR results are expressed as a ratio of the transcript of interest and the housekeeping gene on the whole piece of tissue, the reduction of LFABP and IFABP mRNA levels observed in adult CD patients is likely due to histological changes. Healthy tissue contains a long layer of epithelial cells and a limited* lamina propria*, whereas in severe enteropathy it is just the opposite, a reduced epithelial layer and a larger* lamina propria*. Consequently, to evaluate whether the change in the histology may explain the results obtained, we used villin as housekeeping gene. It is worth noting that villin specificity is such that its promoter has been used for transgenic expression of different proteins limited to the enterocyte [47]. The epithelium loss in duodenal samples from untreated CD patients was accompanied by a reduction in the relative expression of villin. The analysis of LFABP and IFABP mRNA expression using villin as housekeeping gene showed higher levels of LFABP and IFABP transcripts in CD samples compared to non-CD controls, reaching statistical significance for IFABP (Figure 6). This would be suggesting an upregulation in FABPs expression within the enterocytes, as described in the renal ischemic injury for LFABP [48].

Taken together, we observed that the expression of LFABP and IFABP is limited to fully differentiated enterocytes in healthy tissue whereas in active CD they are also expressed in immature enterocytes from the crypts. Though the mRNA analysis using villin as housekeeping gene showed that FABPs expression is increased within enterocytes in CD enteropathy, the total expression in the tissue, referred to *β*-actin, was diminished. In accordance with these findings, Simula et al. [49], using quantitative proteomic analysis, found lower levels of LFABP (FABP1) and IFABP (FABP2) in duodenal samples from active CD patients compared to healthy small intestine, using vinculin as normalizing protein. Considering the multiple biological roles of FABPs in the intestinal mucosa, the lower expression of FABPs may have pathological effects. One of the possible mechanisms involved is through the PPAR*γ* signaling pathway. PPAR*γ* is a ligand-activated nuclear receptor which plays multiple roles in metabolism, cellular proliferation, differentiation, and immune response [50]. Particularly, PPAR*γ* performs relevant biological functions in the intestinal mucosa, where it also contributes to anti-inflammatory and regulatory effects [51]. The interaction of LFABP with PPAR*α* and PPAR*γ* has already been described in hepatocytes [52], and LFABP's function as a mediator in PPAR activity in the nucleus has been shown. Taking this into account, we can hypothesize that LFABP and IFABP may modulate the function of PPAR*γ* in enterocytes, and thus changes in FABPs expression may affect PPAR*γ* activation. Interestingly, downregulation of FABPs and PPAR*γ* was found in small intestine of CD patients [49], which may explain some of the alterations observed in signaling pathways in active CD. LFABP and IFABP knock-out mice have shown alterations in mucosal lipid metabolism, with differential lipid assimilation in each knock-out type [53]. In this context, it is possible to speculate that lipid metabolism may be altered in this pathology as well. Further studies will be carried out to asses this hypothesis. In this work, we showed changes in the pattern of expression of LFABP and IFABP in proximal small intestine in severe enteropathy. These changes are likely associated with the histological changes observed in the intestinal mucosa in active CD and alterations in enterocyte differentiation. In conclusion, these findings suggest that changes in FABPs expression may have a relevant influence in the control of the immune response as well as other important functions in the intestinal mucosa, which altogether may contribute to the pathogenic mechanism of damage in active CD.

## Supplementary Material

Supplementary Figure 1. Isotypic control for immunofluorescence microscopy experiments. Con-focal fluorescence microscopy analysis using unimmunized rabbit serum, dilution1/20 (detected with a secondary Alexa 488, green) and nuclei stained with propidium iodide (red). Representative staining in duodenal sections of non-CD control (A and B) and CD patient at diagnosis (C) (Magnification 63X + 1.7 zoom). Representative staining shows that the unimmunized serum does not react with any of the proteins present in the control or CD samples.

## Abbreviations

CD: Celiac disease
GFD: Gluten-free diet
IBD: Intestinal bowel disease
FABPs: Fatty acid binding proteins
LFABP: Liver fatty acid binding protein
IFABP: Intestinal fatty acid binding protein.
