# Supplementary figures and images for: Expression Pattern of Fatty Acid Binding Proteins in Celiac Disease Enteropathy
Source: Mediators Inflamm. 2015 Aug 5;2015:738563. doi: 10.1155/2015/738563 (PMC4540995; doi:10.1155/2015/738563)

Figure Supplementary 1

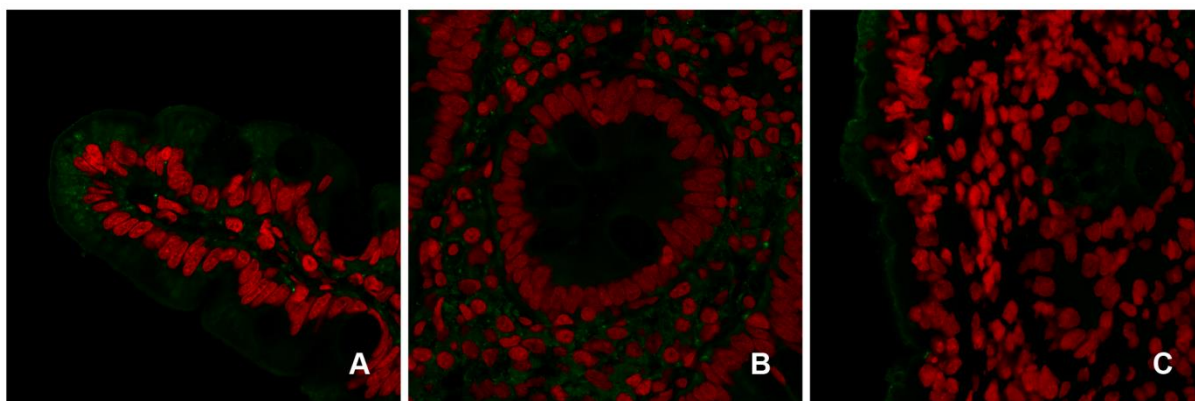

Supplement: Supplementary file 1 — Supplementary Figure 1. Isotypic control for immunofluorescence microscopy experiments. Con-focal fluorescence microscopy analysis using unimmunized rabbit serum, dilution1/20 (detected with a secondary Alexa 488, green) and nuclei stained with propidium iodide (red). Representative staining in duodenal sections of non-CD control (A and B) and CD patient at diagnosis (C) (Magnification 63X + 1.7 zoom). Representative staining shows that the unimmunized serum does not react with any of the proteins present in the control or CD samples. [file 738563.f1.pdf]
